# Intraoperative Detection of Distal True Lumen Collapse and Endovascular Salvage Following Frozen Elephant Trunk Repair for Acute Stanford Type A Intramural Hematoma

**DOI:** 10.3400/avd.cr.26-00026

**Published:** 2026-07-30

**Authors:** Norimasa Haijima, Mikihiko Kudo, Satoru Murata, Takuma Aoki, Hideyuki Shimizu, Takashi Kunihara

**Affiliations:** 1 Department of Cardiovascular Surgery, National Hospital Organization Saitama Hospital, Wako, Saitama, Japan; 2 Department of Cardiovascular Surgery, Keio University Hospital, Tokyo, Japan; 3 Department of Cardiac Surgery, Jikei University Hospital, Tokyo, Japan

**Keywords:** frozen elephant trunk, true-lumen collapse, thoracic endovascular aortic repair

## Abstract

A 76-year-old female Jehovah’s Witness presented with an acute Stanford type A intramural hematoma. To avoid transfusion, she underwent ascending aortic replacement with a frozen elephant trunk and total fenestration. During chest closure, she developed sudden bilateral lower-limb ischemia, with regional oxygen saturation declining from 70% to 40%. Transesophageal echocardiography confirmed patency of the frozen elephant trunk and revealed rapid collapse of the distal true lumen beyond the stented segment. Emergent angiography demonstrated severe true-lumen compression. Thoracic endovascular aortic repair with distal bare-metal stent support was performed urgently, restoring flow. Stent deployment began 50 min after ischemia onset, and complete reperfusion was achieved 82 min after onset. The patient was discharged on postoperative day 17 without neurologic or ischemic sequelae.

## Introduction

Acute Stanford type A aortic dissection or intramural hematoma (IMH) frequently requires urgent surgery, and perioperative transfusion is common. In Jehovah’s Witness patients, however, refusal of allogeneic blood products can particularly constrain operative planning and rescue strategies. Bloodless repair has been reported only in selected settings with meticulous multidisciplinary preparation.^1)^ In this context, limiting surgical extent and shortening cardiopulmonary bypass (CPB) time may be prioritized to complete lifesaving repair without transfusion, yet residual distal malperfusion can still occur. Distal true-lumen collapse is a key mechanism of lower-body ischemia in acute type A disease, and conventional CPB strategies may not always restore distal perfusion. Staged or adjunct endovascular repair may be required even after the entry resection.^2,3)^ Endovascular approaches combining proximal stent-graft coverage with distal bare-metal stent support have been proposed to expand the true lumen and mitigate malperfusion.^4)^ Furthermore, technical refinements such as the PETTICOAT–snowshoe concept may reduce distal stent graft-induced new entry.^5)^ We report a Jehovah’s Witness case with acute Stanford type A IMH and a zone 2 ulcer-like projection who underwent ascending aortic replacement with a frozen elephant trunk (FET) and total fenestration under a strict bloodless protocol. Despite apparently successful central repair, sudden distal true-lumen collapse developed during chest closure. Prompt intraoperative detection using lower-limb oxygen saturation monitoring and Transesophageal echocardiography (TEE) enabled immediate endovascular salvage.

## Case Report

A 76-year-old Jehovah’s Witness woman (150 cm, 41.7 kg; BSA 1.4 m^2^) was transferred for emergency surgery due to an acute Stanford type A IMH with a zone 2 ulcer-like projection (ULP). She had experienced fluctuating back pain since January 28, 2026, which acutely intensified on February 2. Upon arrival, she was hemodynamically stable (BP 139/83 mmHg, HR 100 bpm) with no neurological deficits, pulse deficits, cardiac tamponade, or signs of cerebral or myocardial ischemia. Given the persistent symptoms, the zone 2 ULP, and an IMH thickness exceeding 10 mm, we proceeded with emergency surgery despite the elevated risks associated with a strict bloodless strategy. The patient also strongly requested operative intervention.

The patient had signed a formal declaration refusing allogeneic blood products (red blood cells, fresh frozen plasma, platelets, and whole blood) but consented to intraoperative cell salvage and the use of specific blood fractions. Preoperative hemoglobin was 13.2 g/dL, and creatinine was 0.62 mg/dL. Contrast-enhanced computed tomography (CT) revealed an IMH involving the ascending aorta (diameter: 46.7 mm; IMH thickness: 17.2 mm) and a small dorsal ULP in zone 2 (**Fig. 1A** and **1B**). No dissection-related extension was identified distal to the celiac axis on preoperative CT.

**Fig. 1:**
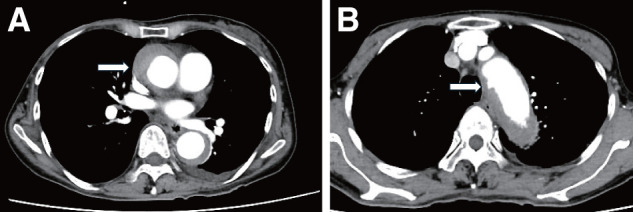
Preoperative computed tomography. (**A**) Axial image of the ascending aorta showing an intramural hematoma with a thrombosed false lumen; arrow indicates the crescentic wall thickening. (**B**) Axial image at the aortic arch demonstrating a small ulcer-like projection on the dorsal side around zone 2; arrow indicates the ulcer-like projection.

### Surgical procedure

Since entry resection would have required distal transection in zone 3 with supra-aortic branch reconstruction, we reasoned that a conventional partial arch replacement might not minimize blood loss in this patient. A zone 3 transection would have prolonged circulatory arrest time and increased the number of anastomoses, thereby elevating the risk of bleeding. Consequently, to maintain a bloodless strategy while achieving central repair, we performed ascending aortic replacement combined with a FET and total fenestration.

Under moderate hypothermic circulatory arrest (27°C) and selective cerebral perfusion, ascending aortic replacement was performed with antegrade perfusion from the ascending aorta. A longitudinal entry tear was identified spanning zones 2–3. The FET was intended to cover and seal the arch entry/ULP. An open stent graft (Frozenix 31 × 90 mm; Japan Lifeline, Tokyo, Japan) was deployed in zone 0. Total fenestration was then created by island-shaped resection of the 3 supra-aortic branch orifices using Metzenbaum scissors. The outer circumference of the fenestrated segment was secured continuously to the native aorta and the FET using 4-0 Prolene (Ethicon, Raritan, NJ, USA) to ensure arch branch perfusion. Weaning from CPB was uneventful. The total operative time was 5 hrs and 43 min, with an estimated blood loss of 956 mL.

### Complication and management

During chest closure, approximately 10 min after protamine administration, bilateral pedal oxygen saturation became undetectable, and regional oxygen saturation (In-Vivo Optical Spectroscopy [INVOS]) in both lower legs dropped from 70% to 40%. TEE confirmed FET patency without evidence of kinking but demonstrated abrupt, severe narrowing of the distal true lumen beyond the stented segment at the level of the descending thoracic aorta. These findings suggested acute lower-body malperfusion caused by distal true-lumen collapse rather than FET obstruction.

Emergency thoracic endovascular aortic repair (TEVAR) was immediately performed via the right femoral artery. Aortography revealed near-absent true-lumen perfusion due to severe compression by a rapidly thrombosing false lumen (**Fig. 2A**). Angiography showed no evidence of fenestration-related endoleak, angiographic flow from the fenestration into the ULP, or a new entry suggestive of distal stent graft-induced new entry (d-SINE). A thoracic stent graft (GORE TAG; W. L. Gore & Associates, Newark, DE, USA) was deployed distal to the FET, followed by 2 distal bare-metal stents (Cook Medical; Bloomington, IN, USA) extending to the terminal aorta (PETTICOAT strategy). This distal scaffold support was necessary to restore true-lumen caliber and maintain visceral perfusion, as the compression involved the abdominal aorta. An additional thoracic stent graft (Cook Medical) was placed up to the Th8 level using the “snowshoe” concept. This additional covered stent aimed not only to extend coverage but also to disperse distal stent-edge stress within the bare stent scaffold, reducing the risk of d-SINE.

**Fig. 2:**
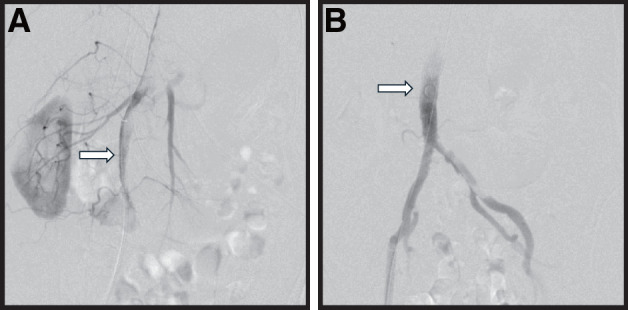
Emergent intraoperative angiography during endovascular rescue. (**A**) Abdominal aortography before endovascular intervention demonstrating severely compromised true-lumen perfusion with poor distal opacification; arrow indicates the markedly narrowed true-lumen segment. (**B**) Completion angiography after thoracic endovascular aortic repair with distal bare-metal stent support (PETTICOAT strategy) demonstrating restoration of antegrade flow to the distal aorta; arrow indicates improved true-lumen perfusion.

Final angiography confirmed resolution of the compression and restoration of distal flow (**Fig. 2B**); INVOS values recovered to 70%. TEE confirmed re-expansion of the true lumen and antegrade flow. The intervention was initiated 50 min after the ischemic event, with complete reperfusion achieved 82 min later.

### Outcomes

Perioperative management followed a strict bloodless protocol. Intraoperative cell salvage was utilized, including the return of salvaged blood via the bypass circuit. The nadir hemoglobin was 6.8 g/dL. No allogeneic products were administered; instead, Factor XIII concentrate (Fibrogammin P, 4 vials) was used. Postoperatively, peak creatinine reached 3.16 mg/dL but improved to 0.98 mg/dL without renal replacement therapy. The patient was discharged ambulatory on postoperative day 17 with a hemoglobin of 9.8 g/dL. Follow-up CT, including sagittal imaging aligned with the curvature of the aortic arch, demonstrated patent arch branches through the fenestrated segment, no obvious fenestration-related endoleak, no significant narrowing around the FET anastomotic region, complete disappearance of the false lumen, and no evidence of FET kinking or d-SINE (**Fig. 3A**–**3C**).

**Fig. 3:**
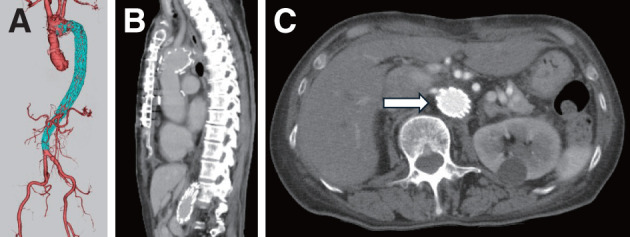
Postoperative computed tomography after endovascular salvage. (**A**) Three-dimensional reconstruction demonstrating the repaired thoracic and abdominal aorta with the deployed endovascular devices. (**B**) Sagittal computed tomography image aligned with the curvature of the aortic arch, showing the postoperative configuration of the fenestrated frozen elephant trunk. The image demonstrates preserved luminal continuity without obvious fenestration-related endoleak or significant narrowing around the FET anastomotic region. (**C**) Axial image at the renal artery level showing expansion of the true lumen with preserved renal perfusion. Arrow indicates the expanded true lumen and the endovascular stent structure.

## Discussion

This case illustrates a particularly high-risk clinical scenario: acute Stanford type A IMH with a zone 2 ULP in a Jehovah’s Witness patient managed under a strict bloodless protocol. In this setting, prolonged bleeding, extended CPB duration, and delayed rescue interventions are poorly tolerated. Even when proximal repair and weaning from CPB appear uneventful, distal aortic hemodynamics can shift abruptly, and delayed-onset lower-body malperfusion may rapidly become life-threatening.

### Rationale for the selected arch strategy under bloodless constraints

Although bloodless arch repair in Jehovah’s Witness patients has been reported with meticulous planning, such cases remain inherently vulnerable to unexpected complications that may necessitate additional invasive rescue procedures.^1)^ In our patient, operative planning prioritized completion of lifesaving central repair without transfusion by minimizing CPB time, circulatory arrest duration, and the number of anastomoses. Although entry resection is a fundamental principle in the management of acute type A pathology, conventional distal transection in zone 3 would have required supra-aortic branch reconstruction, prolonged hypothermic arrest, and increased bleeding risk. Consequently, we selected ascending aortic replacement with zone 0 FET deployment and total fenestration. This approach served as a practical blood-sparing compromise, allowing entry coverage while avoiding more time-consuming arch reconstruction. Fenestrated FET concepts, which simplify branch management, have shown encouraging results in acute type A dissection.^6,7)^ In the present case, total fenestration of the 3 supra-aortic branches was created manually, and the outer circumference was secured continuously to the native aorta and the FET to maintain branch perfusion.

### Mechanism of distal true-lumen collapse

Despite an initially smooth course after CPB, the patient developed sudden bilateral lower-limb ischemia during chest closure. This pattern suggests an abrupt change in distal perfusion mechanics, specifically, rapid collapse of the distal true lumen. In acute type A disease, true-lumen collapse is a recognized mechanism of malperfusion, whereby an enlarging, pressurized, or thrombosing false lumen compromises the true-lumen caliber.^2)^ In the present case, the most likely mechanism was rapid false-lumen pressurization and subsequent thrombosis after proximal entry closure. We hypothesize that, once the proximal entry/ULP had been excluded by the FET, residual retrograde false-lumen flow from distal re-entry sites persisted transiently while proximal outflow had been eliminated. Subsequent protamine administration and progression of thrombosis likely accelerated false-lumen organization, thereby increasing external compression on the distal true lumen.

Importantly, alternative mechanisms were not supported by the available findings. TEE confirmed FET patency without evidence of kinking. Emergency angiography demonstrated no fenestration-related endoleak, no flow into the ULP, and no evidence of d-SINE. Furthermore, iatrogenic propagation related to femoral perfusion was unlikely because the index operation was performed with antegrade perfusion from the ascending aorta. Postoperative CT further confirmed the absence of kinking, endoleak, or d-SINE. Accordingly, rapid false-lumen thrombosis with secondary true-lumen compression remains the most plausible explanation in this case.

### Real-time recognition and diagnosis

A key lesson from this case is the importance of immediate recognition. The simultaneous loss of pedal oxygen saturation, marked decline in lower-limb INVOS, and TEE evidence of abrupt true-lumen narrowing prompted immediate angiographic confirmation. TEE was particularly valuable in distinguishing distal true-lumen collapse from mechanical obstruction of the FET itself. This distinction is critical because the rescue strategy for distal collapse differs fundamentally from that for proximal graft failure. After intervention, TEE confirmed true-lumen re-expansion and restoration of antegrade flow, supporting the hemodynamic effectiveness of the endovascular rescue.

### Endovascular salvage and the bloodless strategy

When distal malperfusion emerges after proximal repair, conventional measures may fail to restore perfusion if true-lumen collapse is advanced. Previous reports suggest that adjunct or staged TEVAR may be required even after FET repair to address residual malperfusion.^3)^ In this case, TEVAR was initiated within 50 min of ischemia recognition, and complete reperfusion was achieved 82 min after onset. This rapid timeline was especially important in a no-transfusion setting. Reopening the chest or reinitiating CPB would have imposed substantial additional hemorrhagic risk. In contrast, endovascular rescue allowed prompt restoration of distal flow without further extensive open dissection or additional arch anastomoses.

### Role of PETTICOAT and the snowshoe concept

The PETTICOAT concept, combining proximal stent-graft coverage with distal bare-metal stent support, is intended to expand the true lumen and improve distal perfusion.^4)^ In cases dominated by true-lumen collapse, the bare-metal extension provides an essential scaffold for the compressed lumen beyond the covered segment. Because true-lumen compression in our patient extended into the abdominal aorta, bare-metal stents were deployed to the terminal aorta to support the distal true lumen and maintain visceral and lower-body perfusion.

Furthermore, the PETTICOAT–snowshoe method has been proposed to reduce distal stent graft-induced new entry while maintaining true-lumen support.^5)^ This point deserves emphasis in the present case. The principal role of the bare-metal stents was to expand and support the distal true lumen. In contrast, the additional covered stent placed within the bare-metal scaffold was intended to disperse distal stent-edge stress and reduce the likelihood of d-SINE. Thus, in this acute rescue setting, the snowshoe concept was applied not only as a remodeling adjunct but also as a preventive strategy against new distal injury created by the rescue itself. This combination strategy achieved immediate restoration of flow without neurologic or ischemic sequelae.

### Clinical implications

This case offers 2 practical implications. First, bloodless repair for acute type A pathology should not be understood simply as a less invasive operation, but rather as a strategy that must anticipate potential rescue failure modes in advance. Second, when fenestrated FET is selected to simplify arch repair, clinicians must remain vigilant for abrupt changes in distal hemodynamics even after apparently successful central repair. Continuous lower-body monitoring, intraoperative TEE, early angiographic confirmation, and immediate endovascular capability should therefore be integrated into the operative plan whenever possible.

### Limitations

This report describes a single case and cannot establish an optimal rescue configuration or definitively prove the hemodynamic mechanism. Although intraoperative TEE, angiography, and postoperative CT did not support FET kinking, fenestration-related endoleak, d-SINE, or perfusion-related propagation, the proposed mechanism remains an inference based on the totality of findings rather than on direct false-lumen pressure measurement. In addition, the favorable early result does not eliminate the need for continued imaging follow-up.

## Conclusion

Intraoperative distal true-lumen collapse can occur abruptly after fenestrated FET repair for acute type A IMH and may cause sudden, profound lower-body malperfusion. In a Jehovah’s Witness patient managed under a strict bloodless protocol, rapid recognition using lower-limb oxygen saturation monitoring and TEE, followed by immediate angiographic confirmation, enabled prompt endovascular salvage. TEVAR was initiated within 50 min after ischemia onset, and complete reperfusion was achieved 82 min after onset. This led to recovery without neurologic or ischemic sequelae and allowed discharge on postoperative day 17. When surgical extent is intentionally minimized to preserve bloodless feasibility, a predefined hybrid rescue pathway should be incorporated into the operative strategy.
